# Editorial perspective: Bayesian statistical methods are useful for researchers in child and adolescent mental health

**DOI:** 10.1111/jcpp.13662

**Published:** 2022-07-11

**Authors:** Erling W. Rognli, Rune Zahl‐Olsen, Sondre Sverd Rekdal, Asle Hoffart, Thomas Bjerregaard Bertelsen

**Affiliations:** ^1^ Department of Child and Adolescent Mental Health Services Akershus University Hospital Lørenskog Norway; ^2^ Department of Child and Adolescent Mental Health Sørlandet Hospital Kristiansand Norway; ^3^ Research Institute of Modum Bad Psychiatric Hospital Vikersund Norway; ^4^ Department of Psychology University of Oslo Oslo Norway; ^5^ Department of Clinical Child and Adolescent Psychology University of Bergen Bergen Norway

## Abstract

Bayesian statistical approaches offer nuanced, detailed, and intuitive analyses, even with small sample sizes. Although these qualities are highly relevant for researchers in child and adolescent mental health, Bayesian methods are still quite rarely employed. This editorial perspective will briefly describe what is different about Bayesian statistical methods, discuss some of the ways they may benefit research in our field, and provide an introduction to how Bayesian statistics are employed in practical research.

## What is useful about Bayesian methods?

Researchers in child and adolescent mental health often wish to use statistical methods to answer questions like ‘How certain can we be that treatment A is more efficacious than treatment B?’ or ‘How strong is the evidence for a non‐trivial positive association between these two variables?’. Different sorts of statistical analysis can answer different sorts of questions, and it is important to ask the sort of question that can actually be answered by your analysis (Lakens, 2021). Bayesian methods can give direct answers to questions such as those quoted above, in the form of probabilities such as 72% or 98%, given our prior knowledge and what we observed in our study. The reason for this is that the output of Bayesian methods are probability distributions for model parameters, conditional on the data and our assumptions. We can use these probability distributions, properly called posterior distributions, to make directly interpretable probability statements about any model parameters of interest (e.g. 75% probability of *β* > 0, Kruschke, 2015).

A very useful property of this form of inference is that it is equally applicable for assessing the strength of evidence for no association or effect as it is for the opposite. Bayesian methods therefore simplify the study and reporting of null findings. A posterior distribution concentrated around zero represents evidence for a non‐existent or very small effect just as much as a posterior concentrating on a large effect would be evidence of that.

### Good small sample performance

In a lot of child and adolescent mental health research, it is difficult to get large sample sizes, and underpowered studies are common. More data will of course always give more information than less data of the same kind, whatever the analytic method, but Bayesian methods have many favorable properties when sample sizes are small. Bayesian estimation does not rely on the asymptotic properties of large samples, and will perform well with small sample sizes as long as one is careful about making realistic assumptions (McNeish, 2016).

Further, the way Bayesian methods can yield graded evidence allows us to get more information from small studies. For example, say we conduct a small treatment study, and find that 72% of the posterior distribution for the difference between the groups lies above our threshold for a clinically significant difference – in other words a posterior probability of 72% for such a difference. While that is not a level of certainty that would justify drawing clinical implications, it is still more likely than not that there is actually a difference. Such a finding would thus justify collecting more data to increase the precision of our estimates, and hence our certainty.

### Sequential data analysis

If we decide to collect more data in such a situation, it is easy to incorporate it in a Bayesian analysis. The sampling intentions of the researcher does not affect a Bayesian analysis the same way as it does null‐hypothesis significance testing. We can in fact keep sampling new cases and use them to update our analysis until we achieve a satisfactory level of precision in the parameter estimates (Kruschke, 2015). Rather than having to plan our sample size based on perhaps insufficient information, we can run the study until we are satisfied with the certainty of our conclusions, or until we run out of patients or funding. A recent study within our field of research gives reason to believe that in many cases the researchers would have been able to conclude with Bayesian methods after collecting a smaller sample, and could have terminated their data collection earlier than they did (Bertelsen, Hoffart, Rekdal, & Zahl‐Olsen, 2022). Participation in research represents an investment of time and effort from participants, and research requires allocation of limited resources from research funders; as researchers, we should not expand either of them needlessly.

### Priors – an advantage, not a problem

When conducting a Bayesian analysis, we combine a prior distribution for the parameters with a likelihood for the data once we observe them to get the posterior distribution. Priors are probability distributions assigned to all model parameters independently of the observed data. They encode in a transparent way what assumptions we are making about the parameter values, as long as we report our priors when publishing our findings. Some who are unfamiliar with Bayesian statistics feel uneasy about letting information external to the data inform the analysis in this way. However, we always make assumptions when conducting statistical analyses, Bayesian or not, and we always actually know something about the possible range of parameter values (Gelman, Simpson, & Betancourt, 2017).

In a regression analysis with standardized predictors and outcomes in child and adolescent mental health, we know with practical certainty that no coefficient will be greater than ±4.0, as in most cases it would be sensational to find a coefficient of ±2.0 or larger. This information can and should be included in the analysis; our posterior distribution would otherwise understate what we have actually learned from gathering and analyzing our data. In our view, priors are one of the many advantages of Bayesian analysis, not a difficulty that we need to bypass. Having priors allows us to integrate previous findings from our own studies or the literature directly in the analysis in a transparent way: When setting up our analysis, we could use the posterior from a previous study or the distribution of effects found in a meta‐analysis to inform our choice of prior distributions. Using such informative priors can protect against spurious findings (Pedroza, Han, Truong, Green, & Tyson, 2018), but it will also ensure that the uncertainty in our parameter estimates is not inflated by excluding relevant information from the analysis.

## How to get started with Bayesian methods?

Starting to use Bayesian statistics can nevertheless seem like a large hurdle for a researcher not familiar with it. Learning new methodology always requires an investment of time, but there are many useful resources available. We will here give a brief sketch of a recommended Bayesian statistical workflow (Gelman et al., 2020), and introduce core concepts. In the online supplement to this editorial perspective, we have also provided runnable R‐code demonstrating a simple Bayesian analysis, which can provide a starting point for the interested reader. We also recommend the more extensive introductory papers by Baldwin and Larson (2017) or by Kruschke and Liddell (2018) as well as the introductory reading list compiled by Etz, Gronau, Dablander, Edelsbrunner, and Baribault (2018).

### The steps of a Bayesian analysis

There are various software implementations of Bayesian statistics. We recommend the Stan platform for model fitting (Stan Development Team, 2019). Stan has a very powerful sampling algorithm with interfaces for common statistical software (R, Stata and Python among others). For R users, the package brms (Bürkner, 2017) is a popular choice, featuring flexible specification of multilevel regression models with syntax resembling the widely used lme4 package, and model fitting done in Stan. Our examples here will mainly refer to brms functionality, but custom models can also be coded directly in the Stan language, allowing for incredible flexibility in specifying complex or novel models.

### Defining a model

The first step in a Bayesian analysis is to define the model, choosing a likelihood for the data and priors for all necessary model parameters. Our choice of likelihood will be informed by our assumptions and knowledge about the data‐generating process we are trying to model. In many applications, we perform some form of regression modeling, assuming our data to be continuous normally distributed variables. This implies a normal distribution for the likelihood. However, if we are concerned about outliers distorting our estimates, we can easily use a Student's *t*‐distribution with heavier tails to achieve a more robust fit. We can also model regressions of dichotomous, ordinal, categorical, or count variables by choosing appropriate distributions.

We then proceed to consider the priors needed for the model parameters, whether they be regression coefficients, error variances, or other parameters. A useful way of evaluating prior choice is to conduct a Prior Predictive Check. We use computer simulation to make draws from the prior distributions, and then draw a value of the outcome variable from the likelihood conditional on the values drawn from the priors, repeating this process thousands of times. Combining these draws approximates the distribution of the outcome variable implied by our priors. If that distribution is missing likely values or ranges, our priors are too restrictive, and if it contains impossible or too many improbable values, we actually know more than we have encoded in our priors. In the latter case, we should change them. In the brms package, there are convenient functions to conduct Prior Predictive Checking.

### Fitting the model and verifying accuracy of computation

After specifying a model with priors, we want to fit it to data, multiplying our prior distribution with the likelihood of the data according to Bayes theorem to derive the posterior distribution. Usually it is impossible to derive the posterior distribution analytically, and we must approximate it using Markov Chain Monte Carlo (MCMC) methods, which is what our statistical software allows us to do.

After fitting a model, we must first verify that our use of MCMC was successful. Stan has sensitive diagnostics to assist us in this, which are available in all software interfaces. If there are signs of computational failure, the different chains of the algorithm have not converged to the same posterior, or the number of effectively independent samples from the posterior is insufficient, we should not trust that the algorithm has validly approximated the full posterior distribution. If so, we need to sort out our computational problems before moving on with our analysis.

### Checking model fit

Satisfied that the computation was successful, we proceed to consider whether the model fits the data. To do this, we conduct a Posterior Predictive Check. We draw samples from the distribution of the dependent variable that is implied by our fitted model, given our predictors. If our model fits well, this distribution should resemble the actual distribution of our dependent variable. If it does not, we must consider what it is about our data that our model is failing to capture, and perhaps revise the model.

### Inspecting the posterior distribution

With a reasonably well‐fitting model, we can use the posterior distributions of the model parameters for statistical inference. We can plot or summarize the posterior distributions in various ways. The mean of the posterior is our best point estimate, given an approximately normal posterior, but we should always report and look for the uncertainty in the estimates. We can calculate the proportion of the posterior above or below some value, or report credible intervals. These are intervals containing some proportion of the posterior distribution. They can be interpreted as the range where the true parameter value can be found with the corresponding probability. It has been shown that classical confidence intervals are often incorrectly interpreted as Bayesian credible intervals (Hoekstra, Morey, Rouder, & Wagenmakers, 2014), suggesting that credible intervals have very intuitive interpretations. Which intervals to report depends on the level of certainty required, credible intervals such as 50%, 66%, 89%, 90%, or 95% have been used. Reporting several intervals can be informative to the reader. Note that 95% credible intervals are known to be unreliable without many samples from the tails of the posterior distribution, so if they are to be reported, care must be made that the effective sample size (ESS) is sufficient. Exactly how many samples are sufficient depends on how the effective sample size is calculated. Recently developed methods allow for estimating the tail ESS separately, and when using these estimators (implemented as standard in recent versions of brms and other Stan interfaces) a tail ESS of at least 400 for each parameter is considered adequate (Vehtari, Gelman, Simpson, Carpenter, & Bürkner, 2021). When using older estimators of the ESS, 10,000 for the overall sample size has been recommended for reliable use of the 95% interval (Kruschke, 2018).

### Comparing to other models

We can also compare our model to other models fitted to the same data, if that is relevant to our research question. We can use Bayes Factors to quantify the level of evidence provided by the data for one model over another, for instance for a regression with a treatment by time interaction compared with one without (Rouder, Speckman, Sun, Morey, & Iverson, 2009). We can also use different forms of cross‐validation to estimate how well our models would fit to another sample than the one we collected (Vehtari, Gelman, & Gabry, 2017). Cross‐validation is particularly useful if we are concerned that a complex model may overfit – representing particularities of the sample rather than substantial features of what we are trying to model. Efficient cross‐validation is available in the brms package.

## Conclusions

In summary, Bayesian methods are useful, and align well with the kinds of questions we wish to answer with our analyses. We hope this introduction will encourage many readers to try their hand at learning Bayesian analysis, as well as assist them when evaluating such analyses reported in this study.

## Supporting information


Supporting information

Supporting information

Supporting information
