# Supplementary material for: Editorial perspective: Bayesian statistical methods are useful for researchers in child and adolescent mental health
Source: J Child Psychol Psychiatry. 2022 Jul 11;64(2):339–42. doi: 10.1111/jcpp.13662 (PMC10084248; doi:10.1111/jcpp.13662)
Supplement: Supplementary file 1 — Supporting information [file JCPP-64-339-s001.docx]

# DEMONSTRATION OF A BAYESIAN ANALYSIS

# This script is supplementary to "Editorial Perspective: Bayesian statistical

# methods are useful for researchers in child and adolescent mental health", by

# Rognli, Zahl-Olsen, Rekdal, Hoffart, & Bertelsen (doi: 10.1111/JCPP.13662),

# and demonstrates a simplified Bayesian analysis of simulated data, relevant to

# the field of child and adolescent mental health.

# This version of the script is written for running the example analysis on your

# own computer. It will probably work best when run within the Rstudio IDE.

# Results may vary somewhat from those described due to differences in hardware.

# By setting different random seeds (or not setting a specific seed), you can

# see how much the specific results will vary only due to sampling.

# First of all, if we do not have the necessary packages installed, we must

# install and load them.

if (!require('simstudy')) install.packages('simstudy')

if (!require('brms')) install.packages('brms')

if (!require('tidyr')) install.packages('tidyr')

if (!require('dplyr')) install.packages('dplyr')

if (!require('bayestestR')) install.packages('bayestestR')

library(posterior); library(bayesplot) # these come with brms

# `simstudy` is a package for simulating study datasets, and lets us easily

# simulate some data from a two-group treatment trial with four measurement

# points and a modest sample size. We assume standardized variables. We first

# set the random seed in R to get predictable results in this example

# simulation.

set.seed(426)

time_coef <- .1 # a small effect of time across both groups

treat_time_coef <- .2 # the standardized effect size of treatment for each measurement point

res <- .7 # residual variance

data_definition <-

defData(varname = 'trt_grp', dist = 'binary', formula = .5) %>%

defData(varname = 't0', dist = 'normal', formula = 0, variance = 1) %>%

defData(varname = 't1', dist = 'normal',

formula = 't0 - (..time_coef + ..treat_time_coef * trt_grp)', variance = res) %>%

defData(varname = 't2', dist = 'normal',

formula = 't0 - 2 * (..time_coef + ..treat_time_coef * trt_grp)', variance = res) %>%

defData(varname = 't3', dist = 'normal',

formula = 't0 - 3 * (..time_coef + ..treat_time_coef * trt_grp)', variance = res)

# We generate a dataset with a small N and pivot it to long format for a

# multilevel analysis.

N <- 52

d <- genData(N, data_definition) %>%

pivot_longer(

cols = t0:t3,

names_to = 'time',

values_to = 'y',

names_prefix = 't',

names_transform = list(time = as.numeric))

# We specify a model using standard multilevel modeling syntax with varying

# intercepts (random effects) and a treatment by time interaction.

model <- brmsformula(y ~ (1|id) + time * trt_grp, family = gaussian(link="identity"))

# We first check the model using the `get_prior` function to see which

# parameters may need priors.

get_prior(model, data = d)

# We see that the beta coefficients are assigned uniform priors, which is less

# informative than the prior knowledge we have - uniform priors are flat all the

# way to infinity. Given our small sample size, we need to be particularly

# careful about our priors. Our data is standardized, so we assign normal

# distributions with mean 0 and standard deviation 1.5 to the beta coefficients

# of the model.

model_priors <- set_prior("normal(0, 1.5)", class = 'b')

# To conduct a prior predictive check with brms, we only need to run the model

# with the argument `sample_prior` set to the value `only`. Note that

# compilation of the model and sampling takes a bit of time, depending on your

# system.

prior_check <- brm(model, data = d, prior = model_priors, sample_prior = 'only')

# We then extract outcome variable predictions from the prior model with

# `posterior_predict`, and plot these using the `ppc_stat` function from the

# `bayesplot` package. This gives us a histogram of the prior predictive

# distribution of the mean of the outcome variable. These functions are intended

# for posterior predictive checks, so they require a set of data (y) to plot

# draws (yrep) against. For the y argument (the original data in case of a

# posterior predictive check) we here provide a vector of zeros of the same

# length as the dataset. As our outcome data is standardized, it's reasonable to

# compare the prior predictions of the outcome mean against a mean of zero.

ppc_stat(y = rep(0, nrow(d)),

yrep = posterior_predict(prior_check),

binwidth = 1)

# These priors are certainly not too informative - the prior predictive

# distribution of the mean includes some highly implausible values. Simply

# plotting the priors for the coefficients could probably have told us this in

# the case of this simple model, but for more complex models it is hard to

# understand how priors interact. In such cases, prior predictive checks are

# vital tools for understanding the joint implications of the prior.

# We decide to tighten in our priors for the beta coefficients to a standard

# normal distribution instead. Perhaps we could have represented our prior

# knowledge even better with more work and prior predictive checking, but we

# leave it at this for now.

model_priors <- set_prior("normal(0, 1)", class = 'b')

# Then we fit the model, using the `update` function of brms to reuse the

# compiled model if possible (here it needs recompilation as the prior has

# changed).

fit <- update(prior_check, prior = model_priors, sample_prior = 'no')

# We first need to verify the computation. brms will actually inform us about

# computational problems, but for the sake of the example we call the

# `check_hmc_diagnostics` function from the rstan package on the stanfit object

# contained within the brmsfit object:

rstan::check_hmc_diagnostics(fit$fit)

# All looks well. We can then call summary on the fit to look further at it. As

# we have not run the model for sufficient iterations to get reliable 95%

# credible intervals, we settle for 90% intervals.

summary(fit, prob = .90)

# The indicators of convergence are not satisfactory for some parameters. Rhats

# are >1.09, and although > 400 effective samples would generally considered

# sufficient (minimum 100 per chain), we see that the bulk ESS of these

# parameters are lower than the other parameters. We can also visually assess

# convergence by checking traceplots. Here we want to see what looks like "fat

# caterpillars" indicating that the chains are moving in the same areas. This is

# generally not viable when the model has a large number of parameters.

mcmc_trace(fit, pars = c('b_Intercept', 'b_trt_grp', 'b_time', 'b_time', 'b_time:trt_grp'))

# We can see how the different chains in the top two plots are not mixing as

# well as in the bottom two - the same issue identified by the high Rhats.

# Nevertheless, the chains aren't showing signs of serious issues and there were

# no other diagnostic indicators of more severe computational problems, so this

# problem might be solvable by simply drawing more samples. This model samples

# quite quickly, so we can try to solve the problem by running the chains for a

# bit longer, and see if they converge then. By default, increasing the `iter`

# argument of `brm` increases the warmup and sampling phases equally, so we

# increase the warmup to 1500, and increase the iteration total to 3500,

# doubling the length of the sampling phase, and increasing the warmup by half.

# We again use the function `update` to reuse the compiled Stan model from the

# previous fit.

fit <- update(fit, warmup = 1500, iter = 3500)

# Checking diagnostics and convergence:

rstan::check_hmc_diagnostics(fit$fit)

summary(fit, prob = .90)

# Effective sample sizes now look good for all parameters, both for the bulk and

# the tails of the distribution. All R-hat values are also <1.09, so we are

# satisfied that our computation has approximated the posterior distribution.

# Our next step is to perform posterior predictive checking. We check whether

# the distribution of the outcome that we predict based on our posterior

# distribution is (yrep) is similar to the one we observed (y). We have data for

# different timepoints, so we'd like to check the fit at each point separately,

# as well as for the distribution of the outcome overall. We look at whether the

# distributions have different distributional shapes and importantly whether

# they have different central points or skew/kurtosis. We can start by the

# simple case of calling the `pp_check` function of brms, which will in our case

# consider all the timepoints at once. We specify the `ndraws` argument to 25,

# which means we plot a random sample of 25 posterior predictions rather than

# all of them, as computing and plotting 8000 density lines would take a long

# time, and is not necessary to assess model fit.

pp_check(fit, type="dens_overlay", ndraws=25)

# There is no sign here of the model severely misfitting our data. We would also

# want to plot this for the timepoints separately, to see whether the model is

# misfitting some timepoint more than another. We then need to use the function

# from the `bayesplot` package directly, and use `posterior_predict` to extract

# the necessary `yrep` from our brmsfit object.

draws_y <- posterior_predict(fit, ndraws = 25)

ppc_dens_overlay_grouped(y = d$y,

yrep = draws_y,

group = d$time)

# A violin plot could perhaps work even better to see how well the model is

# fitting the different timepoints, and works better with a large yrep.

draws_y <- posterior_predict(fit, ndraws = 200)

ppc_violin_grouped(y = d$y,

yrep = draws_y,

group = d$time,

y_draw = 'both',

y_jitter = 0.05)

# Judging from these plots our model isn't misfitting these data badly, but

# there are signs that the observed distribution might be different at some

# timepoints than what the model expects. In a real dataset, such observations

# might be clues to important unmodelled sources of variation. We could then

# explore ways of making our model fit these observations better, perhaps

# leading to developing new hypotheses. For now, we are satisfied that the model

# fits reasonably well, and we return to looking at parameter estimates.

# We use a convenient function from the package `bayestestR` to summarise the

# posterior distribution. This allows us to specify a Region of Practical

# Equivalence (ROPE), that is a region of the possible values of the parameter

# that we regard as practically equivalent to no association or effect, and then

# calculates the proportion of the posterior falling within this area.

describe_posterior(fit, ci=0.90, rope_range = c(-.1,.1))

# Looking at the parameter estimates, we see that the fit indicates a likely

# treatment effect (it recovers the generating parameters quite well), but the

# credible intervals are still wide - not surprising given the small sample.

# Note the estimate for the beta coefficient for treatment group (`trt_grp`).

# This is no error but random baseline differences between the groups due to the

# small sample size. To verify that, we could fit the model with a larger

# dataset from the same distribution, and see that the `trt_grp` beta estimate

# would fall to 0 (we tested with N = 400, and it does). Currently, our model is

# simply adjusting for those differences, which is of course a good thing.

# We can also use the `mcmc_plot` function to plot the posterior distributions:

mcmc_plot(fit, type = 'areas')

# We aren't completely satisfied with the certainty of our estimates (around 13%

# of our posterior for the time by treatment interaction is within the ROPE) so

# we decide to collect some more data, to see if that could improve our

# certainty about the findings. We simulate 25 more cases. We need to make the

# id variable of the expanded data consecutive to that of the original dataset

# before pivoting it to long format and joining with the original dataset.

set.seed(303) # reset seed for predictable data simulation results

N_extra <- 25

d_new <- genData(N_extra, data_definition) %>%

mutate(id = seq(N + 1, N + N_extra)) %>%

pivot_longer(

cols = t0:t3,

names_to = 'time',

values_to = 'y',

names_prefix = 't',

names_transform = list(time = as.numeric)) %>%

bind_rows(d)

# And then we refit the model with our expanded dataset, again using the

# "update" function, and the argument "newdata". We save all parameters, because

# we will need them for computing Bayes factors later.

fit_new <- update(fit, newdata = d_new, save_pars = save_pars(all = TRUE))

# We check diagnostics for good measure, and inspect the effective sample sizes

# and R-hats.

rstan::check_hmc_diagnostics(fit_new$fit)

summary(fit_new, prob = .90)

# No convergence problems indicated. Also, the estimate for treatment group is

# now more in line with the generating model. We see that the uncertainty in the

# posterior distributions have decreased. We can illustrate this for the sake of

# our example by plotting the posterior distribution under the original dataset

# and the expanded one in the same plot.

mcmc_areas(tibble('N = 52' = extract_variable(fit, variable = 'b_time:trt_grp'),

'N = 77' = extract_variable(fit_new, variable = 'b_time:trt_grp')))

# We see clearly how the larger dataset decreases the uncertainty about the

# parameter estimate, but that the posterior mean is now slightly overestimating

# the true parameter value. We can use `describe_posterior` again to look closer

# at parameter estimates:

describe_posterior(fit_new, ci=0.90, rope_range = c(-.1,.1))

# Given these data, the treatment by time interaction is highly unlikely to be

# of smaller magnitude than -0.1. To evaluate whether randomization is a good

# explanation of the group difference, we might want to compare our model to one

# with only an effect of time.

model_time <- brmsformula(y ~ (1|id) + time)

fit_time <- brm(model_time, data = d_new, prior = model_priors,

warmup = 1500, iter = 3500, save_pars = save_pars(all = TRUE))

# We can then add leave-one-out crossvalidation model comparison criteria to

# both fit objects, and then compare them using the `loo_compare` function.

fit_new <- add_criterion(fit_new, criterion = 'loo', moment_match = TRUE)

fit_time <- add_criterion(fit_time, criterion = 'loo', moment_match = TRUE)

loo_compare(fit_new, fit_time)

# Which (correctly) favours the model with a treatment effect as likely to

# predict better in a future sample. The difference is non-trivial (>4), but

# there is still some uncertainty, the standard error of the estimate is about

# half that of the difference.

# We can also compute the Bayes Factor:

bayes_factor(fit_new, fit_time)

# Which also indicates that our data gives moderate support for the model with a

# treatment by time interaction over a model with only an effect of time. We are

# left to conclude that the evidence supports a treatment effect, and that it is

# highly unlikely to be smaller than 0.3 standardized units on average over the

# whole observation period. If we were to conduct a replication, a more

# informative prior based on this posterior would be justified.
