# Supplementary material for: Editorial perspective: Bayesian statistical methods are useful for researchers in child and adolescent mental health
Source: J Child Psychol Psychiatry. 2022 Jul 11;64(2):339–42. doi: 10.1111/jcpp.13662 (PMC10084248; doi:10.1111/jcpp.13662)
Supplement: Supplementary file 3 — Supporting information [file JCPP-64-339-s002.html]

Supplemental tutorial to Editorial Perspective: Bayesian statistical methods are useful for researchers in child and adolescent mental health


# Supplemental tutorial to *Editorial Perspective: Bayesian statistical methods are useful for researchers in child and adolescent mental health*

### doi:10.1111/JCPP.13662

#### Rognli, Zahl-Olsen, Rekdal, Hoffart, & Bertelsen

This supplementary script demonstrates a simplified Bayesian analysis of simulated data, relevant to the field of child and adolescent mental health. This version of the document shows the results of the code as well.

## Setting up necessary packages

First of all, if we do not have the necessary packages installed, we must install and load them.

```
if (!require('simstudy')) install.packages('simstudy')
if (!require('brms')) install.packages('brms')
if (!require('tidyr')) install.packages('tidyr')
if (!require('dplyr')) install.packages('dplyr')
if (!require('bayestestR')) install.packages('bayestestR')

library(posterior); library(bayesplot) # these come with brms
```

## Generate simulated data

`simstudy` is a package for simulating study datasets, and lets us easily simulate some data from a two-group treatment trial with four measurement points and a modest sample size. We assume standardized variables. We first set the random seed in R to get predictable results in this example simulation.

```
set.seed(426)

time_coef <- .1 # a small effect of time across both groups
treat_time_coef <- .2 # the standardized effect size of treatment for each measurement point
res <- .7 # residual variance

data_definition <- 
  defData(varname = 'trt_grp', dist = 'binary', formula = .5) %>%
  defData(varname = 't0', dist = 'normal', formula = 0, variance = 1) %>%
  defData(varname = 't1', dist = 'normal', 
          formula = 't0 - (..time_coef + ..treat_time_coef * trt_grp)', variance = res) %>%
  defData(varname = 't2', dist = 'normal', 
          formula = 't0 - 2 * (..time_coef + ..treat_time_coef * trt_grp)', variance = res) %>%
  defData(varname = 't3', dist = 'normal', 
          formula = 't0 - 3 * (..time_coef + ..treat_time_coef * trt_grp)', variance = res)
```

We generate a dataset with a small N and pivot it to long format for a multilevel analysis.

```
N <- 52

d <- genData(N, data_definition) %>%
  pivot_longer(
    cols = t0:t3,
    names_to = 'time',
    values_to = 'y',
    names_prefix = 't',
    names_transform = list(time = as.numeric))
```

## Analysis

### Step 1: Defining the model

We specify a model using standard multilevel modeling syntax with varying intercepts (random effects) and a treatment by time interaction.

```
model <- brmsformula(y ~ (1|id) + time * trt_grp, family = gaussian(link="identity"))
```

### Step 2: Defining and investigating priors

We first check the model using the `get_prior` function to see which parameters may need priors.

```
get_prior(model, data = d)
```

```
##                    prior     class         coef group resp dpar nlpar bound       source
##                   (flat)         b                                               default
##                   (flat)         b         time                             (vectorized)
##                   (flat)         b time:trt_grp                             (vectorized)
##                   (flat)         b      trt_grp                             (vectorized)
##  student_t(3, -0.1, 2.5) Intercept                                               default
##     student_t(3, 0, 2.5)        sd                                               default
##     student_t(3, 0, 2.5)        sd                 id                       (vectorized)
##     student_t(3, 0, 2.5)        sd    Intercept    id                       (vectorized)
##     student_t(3, 0, 2.5)     sigma                                               default
```

We see that the beta coefficients are assigned uniform priors, which is less informative than the prior knowledge we have - uniform priors are flat all the way to infinity. Given our small sample size, we need to be particularly careful about our priors. Our data is standardized, so we assign normal distributions with mean 0 and standard deviation 1.5 to the beta coefficients of the model.

```
model_priors <- set_prior("normal(0, 1.5)", class = 'b')
```

To conduct a prior predictive check with brms, we only need to run the model with the argument `sample_prior` set to the value `only`. Note that compilation of the model and sampling takes a bit of time, depending on your system.

```
prior_check <- brm(model, data = d, prior = model_priors, sample_prior = 'only')
```

```
## Compiling Stan program...
```

```
## Start sampling
```

We then extract outcome variable predictions from the prior model with `posterior_predict`, and plot these using the `ppc_stat` function from the `bayesplot` package. This gives us a histogram of the prior predictive distribution of the mean of the outcome variable. These functions are intended for posterior predictive checks, so they require a set of data (y) to plot draws (yrep) against. For the y argument (the original data in case of a posterior predictive check) we here provide a vector of zeros of the same length as the dataset. As our outcome data is standardized, it’s reasonable to compare the prior predictions of the outcome mean against a mean of zero.

```
ppc_stat(y = rep(0, nrow(d)),
         yrep = posterior_predict(prior_check),
         binwidth = 1)
```

These priors are certainly not too informative - the prior predictive distribution of the mean includes some highly implausible values. Simply plotting the priors for the coefficients could probably have told us this in the case of this simple model, but for more complex models it is hard to understand how priors interact. In such cases, prior predictive checks are vital tools for understanding the joint implications of the prior.

We decide to tighten in our priors for the beta coefficients to a standard normal distribution instead. Perhaps we could have represented our prior knowledge even better with more work and prior predictive checking, but we leave it at this for now.

```
model_priors <- set_prior("normal(0, 1)", class = 'b')
```

### Step 3. Fit model

Then we fit the model, using the `update` function of brms to reuse the compiled model if possible (here it needs recompilation as the prior has changed).

```
fit <- update(prior_check, prior = model_priors, sample_prior = 'no')
```

```
## The desired updates require recompiling the model
```

```
## Compiling Stan program...
```

```
## Start sampling
```

### Step 4. Check computation

We first need to verify the computation. brms will actually inform us about computational problems, but for the sake of the example we call the `check_hmc_diagnostics` function from the rstan package on the stanfit object contained within the brmsfit object:

```
rstan::check_hmc_diagnostics(fit$fit)
```

```
## 
## Divergences:
```

```
## 0 of 4000 iterations ended with a divergence.
```

```
## 
## Tree depth:
```

```
## 0 of 4000 iterations saturated the maximum tree depth of 10.
```

```
## 
## Energy:
```

```
## E-BFMI indicated no pathological behavior.
```

All looks well. We can then call summary on the fit to look further at it. As we have not run the model for sufficient iterations to get reliable 95% credible intervals, we settle for 90% intervals.

```
summary(fit, prob = .90)
```

```
##  Family: gaussian 
##   Links: mu = identity; sigma = identity 
## Formula: y ~ (1 | id) + time * trt_grp 
##    Data: d (Number of observations: 208) 
##   Draws: 4 chains, each with iter = 2000; warmup = 1000; thin = 1;
##          total post-warmup draws = 4000
## 
## Group-Level Effects: 
## ~id (Number of levels: 52) 
##               Estimate Est.Error l-90% CI u-90% CI Rhat Bulk_ESS Tail_ESS
## sd(Intercept)     1.20      0.13     1.00     1.43 1.01      681     1531
## 
## Population-Level Effects: 
##              Estimate Est.Error l-90% CI u-90% CI Rhat Bulk_ESS Tail_ESS
## Intercept        0.05      0.25    -0.36     0.46 1.01      537      802
## time            -0.12      0.06    -0.22    -0.02 1.00     3002     3194
## trt_grp          0.17      0.36    -0.42     0.76 1.02      438     1063
## time:trt_grp    -0.21      0.09    -0.35    -0.06 1.00     2825     2929
## 
## Family Specific Parameters: 
##       Estimate Est.Error l-90% CI u-90% CI Rhat Bulk_ESS Tail_ESS
## sigma     0.72      0.04     0.66     0.80 1.00     3040     3133
## 
## Draws were sampled using sampling(NUTS). For each parameter, Bulk_ESS
## and Tail_ESS are effective sample size measures, and Rhat is the potential
## scale reduction factor on split chains (at convergence, Rhat = 1).
```

The indicators of convergence are not satisfactory for some parameters. Rhats are >1.09, and although > 400 effective samples would generally considered sufficient (minimum 100 per chain), we see that the bulk ESS of these parameters are lower than the other parameters. We can also visually assess convergence by checking traceplots. Here we want to see what looks like “fat caterpillars” indicating that the chains are moving in the same areas. This is generally not viable when the model has a large number of parameters.

```
mcmc_trace(fit, pars = c('b_Intercept', 'b_trt_grp', 'b_time', 'b_time', 'b_time:trt_grp'))
```

We can see how the different chains in the top two plots are not mixing as well as in the bottom two - the same issue identified by the high Rhats. Nevertheless, the chains aren’t showing signs of serious issues and there were no other diagnostic indicators of more severe computational problems, so this problem might be solvable by simply drawing more samples. This model samples quite quickly, so we can try to solve the problem by running the chains for a bit longer, and see if they converge then. By default, increasing the `iter` argument of `brm` increases the warmup and sampling phases equally, so we increase the warmup to 1500, and increase the iteration total to 3500, doubling the length of the sampling phase, and increasing the warmup by half. We again use the function `update` to reuse the compiled Stan model from the previous fit.

```
fit <- update(fit, warmup = 1500, iter = 3500)
```

```
## Start sampling
```

Checking diagnostics and convergence:

```
rstan::check_hmc_diagnostics(fit$fit)
```

```
## 
## Divergences:
```

```
## 0 of 8000 iterations ended with a divergence.
```

```
## 
## Tree depth:
```

```
## 0 of 8000 iterations saturated the maximum tree depth of 10.
```

```
## 
## Energy:
```

```
## E-BFMI indicated no pathological behavior.
```

```
summary(fit, prob = .90)
```

```
##  Family: gaussian 
##   Links: mu = identity; sigma = identity 
## Formula: y ~ (1 | id) + time * trt_grp 
##    Data: d (Number of observations: 208) 
##   Draws: 4 chains, each with iter = 3500; warmup = 1500; thin = 1;
##          total post-warmup draws = 8000
## 
## Group-Level Effects: 
## ~id (Number of levels: 52) 
##               Estimate Est.Error l-90% CI u-90% CI Rhat Bulk_ESS Tail_ESS
## sd(Intercept)     1.20      0.14     0.99     1.44 1.00     1413     2567
## 
## Population-Level Effects: 
##              Estimate Est.Error l-90% CI u-90% CI Rhat Bulk_ESS Tail_ESS
## Intercept        0.04      0.25    -0.37     0.44 1.00     1196     2529
## time            -0.12      0.06    -0.22    -0.02 1.00     5565     5950
## trt_grp          0.19      0.36    -0.40     0.78 1.00     1102     2109
## time:trt_grp    -0.21      0.09    -0.36    -0.06 1.00     5264     5488
## 
## Family Specific Parameters: 
##       Estimate Est.Error l-90% CI u-90% CI Rhat Bulk_ESS Tail_ESS
## sigma     0.72      0.04     0.66     0.80 1.00     6162     5852
## 
## Draws were sampled using sampling(NUTS). For each parameter, Bulk_ESS
## and Tail_ESS are effective sample size measures, and Rhat is the potential
## scale reduction factor on split chains (at convergence, Rhat = 1).
```

Effective sample sizes now look good for all parameters, both for the bulk and the tails of the distribution. All R-hat values are also <1.09, so we are satisfied that our computation has approximated the posterior distribution.

### Step 5. Perform posterior predictive check

Our next step is to perform posterior predictive checking. We check whether the distribution of the outcome that we predict based on our posterior distribution is (yrep) is similar to the one we observed (y). We have data for different timepoints, so we’d like to check the fit at each point separately, as well as for the distribution of the outcome overall. We look at whether the distributions have different distributional shapes and importantly whether they have different central points or skew/kurtosis. We can start by the simple case of calling the `pp_check` function of brms, which will in our case consider all the timepoints at once. We specify the `ndraws` argument to 25, which means we plot a random sample of 25 posterior predictions rather than all of them, as computing and plotting 8000 density lines would take a long time, and is not necessary to assess model fit.

```
pp_check(fit, type="dens_overlay", ndraws=25)
```

There is no sign here of the model severely misfitting our data. We would also want to plot this for the timepoints separately, to see whether the model is misfitting some timepoint more than another. We then need to use the function from the `bayesplot` package directly, and use `posterior_predict` to extract the necessary `yrep` from our brmsfit object.

```
draws_y <- posterior_predict(fit, ndraws = 25)

ppc_dens_overlay_grouped(y = d$y, 
                         yrep = draws_y, 
                         group = d$time)
```

A violin plot could perhaps work even better to see how well the model is fitting the different timepoints, and works better with a large yrep.

```
draws_y <- posterior_predict(fit, ndraws = 200)
ppc_violin_grouped(y = d$y,
                   yrep = draws_y,
                   group = d$time,
                   y_draw = 'both',
                   y_jitter = 0.05)
```

Judging from these plots our model isn’t misfitting these data badly, but there are signs that the observed distribution might be different at some timepoints than what the model expects. In a real dataset, such observations might be clues to important unmodelled sources of variation. We could then explore ways of making our model fit these observations better, perhaps leading to developing new hypotheses. For now, we are satisfied that the model fits reasonably well, and we return to looking at parameter estimates.

### Step 6. Interpretation

We use a convenient function from the package `bayestestR` to summarise the posterior distribution. This allows us to specify a Region of Practical Equivalence (ROPE), that is a region of the possible values of the parameter that we regard as practically equivalent to no association or effect, and then calculates the proportion of the posterior falling within this area.

```
describe_posterior(fit, ci=0.90, rope_range = c(-.1,.1))
```

```
## Summary of Posterior Distribution
## 
## Parameter    | Median |         90% CI |     pd |          ROPE | % in ROPE |  Rhat |     ESS
## ---------------------------------------------------------------------------------------------
## (Intercept)  |   0.04 | [-0.36,  0.44] | 56.96% | [-0.10, 0.10] |    32.14% | 1.004 | 1195.00
## time         |  -0.12 | [-0.22, -0.01] | 96.94% | [-0.10, 0.10] |    39.03% | 1.000 | 5507.00
## trt_grp      |   0.19 | [-0.37,  0.81] | 69.90% | [-0.10, 0.10] |    21.06% | 1.003 | 1099.00
## time:trt_grp |  -0.21 | [-0.35, -0.05] | 98.84% | [-0.10, 0.10] |     9.45% |       |
```

Looking at the parameter estimates, we see that the fit indicates a likely treatment effect (it recovers the generating parameters quite well), but the credible intervals are still wide - not surprising given the small sample. Note the estimate for the beta coefficient for treatment group (`trt_grp`). This is no error but random baseline differences between the groups due to the small sample size. To verify that, we could fit the model with a larger dataset from the same distribution, and see that the `trt_grp` beta estimate would fall to 0 (we tested with N = 400, and it does). Currently, our model is simply adjusting for those differences, which is of course a good thing.

We can also use the `mcmc_plot` function to plot the posterior distributions:

```
mcmc_plot(fit, type = 'areas')
```

### Optional step 7. Refit model on expanded dataset

We aren’t completely satisfied with the certainty of our estimates (around 13% of our posterior for the time by treatment interaction is within the ROPE) so we decide to collect some more data, to see if that could improve our certainty about the findings. We simulate 25 more cases. We need to make the id variable of the expanded data consecutive to that of the original dataset before pivoting it to long format and joining with the original dataset.

```
set.seed(303) # reset seed for predictable data simulation results

N_extra <- 25

d_new <- genData(N_extra, data_definition) %>%
  mutate(id = seq(N + 1, N + N_extra)) %>%
  pivot_longer(
    cols = t0:t3,
    names_to = 'time',
    values_to = 'y',
    names_prefix = 't',
    names_transform = list(time = as.numeric)) %>%
  bind_rows(d)
```

And then we refit the model with our expanded dataset, again using the “update” function, and the argument “newdata”. We save all parameters, because we will need them for computing Bayes factors later.

```
fit_new <- update(fit, newdata = d_new, save_pars = save_pars(all = TRUE))
```

```
## Start sampling
```

We check diagnostics for good measure, and inspect the effective sample sizes and R-hats.

```
rstan::check_hmc_diagnostics(fit_new$fit)
```

```
## 
## Divergences:
```

```
## 0 of 8000 iterations ended with a divergence.
```

```
## 
## Tree depth:
```

```
## 0 of 8000 iterations saturated the maximum tree depth of 10.
```

```
## 
## Energy:
```

```
## E-BFMI indicated no pathological behavior.
```

```
summary(fit_new, prob = .90)
```

```
##  Family: gaussian 
##   Links: mu = identity; sigma = identity 
## Formula: y ~ (1 | id) + time * trt_grp 
##    Data: d_new (Number of observations: 308) 
##   Draws: 4 chains, each with iter = 3500; warmup = 1500; thin = 1;
##          total post-warmup draws = 8000
## 
## Group-Level Effects: 
## ~id (Number of levels: 77) 
##               Estimate Est.Error l-90% CI u-90% CI Rhat Bulk_ESS Tail_ESS
## sd(Intercept)     1.10      0.10     0.94     1.28 1.00     1468     2944
## 
## Population-Level Effects: 
##              Estimate Est.Error l-90% CI u-90% CI Rhat Bulk_ESS Tail_ESS
## Intercept        0.15      0.20    -0.18     0.48 1.00     1281     2197
## time            -0.11      0.05    -0.19    -0.02 1.00     6421     5650
## trt_grp          0.06      0.28    -0.41     0.51 1.00     1166     2484
## time:trt_grp    -0.24      0.07    -0.36    -0.13 1.00     6395     5731
## 
## Family Specific Parameters: 
##       Estimate Est.Error l-90% CI u-90% CI Rhat Bulk_ESS Tail_ESS
## sigma     0.70      0.03     0.64     0.75 1.00     7084     6402
## 
## Draws were sampled using sampling(NUTS). For each parameter, Bulk_ESS
## and Tail_ESS are effective sample size measures, and Rhat is the potential
## scale reduction factor on split chains (at convergence, Rhat = 1).
```

No convergence problems indicated. Also, the estimate for treatment group is now more in line with the generating model. We see that the uncertainty in the posterior distributions have decreased. We can illustrate this for the sake of our example by plotting the posterior distribution under the original dataset and the expanded one in the same plot.

```
mcmc_areas(tibble('N = 52' = extract_variable(fit, variable = 'b_time:trt_grp'),
                  'N = 77' = extract_variable(fit_new, variable = 'b_time:trt_grp')))
```

We see clearly how the larger dataset decreases the uncertainty about the parameter estimate, but that the posterior mean is now slightly overestimating the true parameter value. We can use `describe_posterior` again to look closer at parameter estimates:

```
describe_posterior(fit_new, ci=0.90, rope_range = c(-.1,.1))
```

```
## Possible multicollinearity between b_time:trt_grp and b_time (r = 0.71). This might lead to inappropriate results. See 'Details' in '?rope'.
```

```
## Summary of Posterior Distribution
## 
## Parameter    | Median |         90% CI |     pd |          ROPE | % in ROPE |  Rhat |     ESS
## ---------------------------------------------------------------------------------------------
## (Intercept)  |   0.15 | [-0.20,  0.46] | 76.86% | [-0.10, 0.10] |    32.13% | 1.003 | 1266.00
## time         |  -0.11 | [-0.18, -0.02] | 98.01% | [-0.10, 0.10] |    45.49% | 1.000 | 6433.00
## trt_grp      |   0.06 | [-0.41,  0.51] | 58.67% | [-0.10, 0.10] |    28.72% | 1.001 | 1155.00
## time:trt_grp |  -0.24 | [-0.36, -0.13] | 99.98% | [-0.10, 0.10] |        0% |       |
```

### Step 8. Model comparison

Given these data, the treatment by time interaction is highly unlikely to be of smaller magnitude than -0.1. To evaluate whether randomization is a good explanation of the group difference, we might want to compare our model to one with only an effect of time.

```
model_time <- brmsformula(y ~ (1|id) + time)

fit_time <- brm(model_time, data = d_new, prior = model_priors, 
                warmup = 1500, iter = 3500, save_pars = save_pars(all = TRUE))
```

We can then add leave-one-out crossvalidation model comparison criteria to both fit objects, and then compare them using the `loo_compare` function.

```
fit_new <- add_criterion(fit_new, criterion = 'loo', moment_match = TRUE)

fit_time <- add_criterion(fit_time, criterion = 'loo', moment_match = TRUE)

loo_compare(fit_new, fit_time)
```

```
##          elpd_diff se_diff
## fit_new   0.0       0.0   
## fit_time -6.8       3.3
```

Which (correctly) favours the model with a treatment effect as likely to predict better in a future sample. The difference is non-trivial (>4), but there is still some uncertainty, the standard error of the estimate is about half that of the difference.

We can also compute the Bayes Factor:

```
bayes_factor(fit_new, fit_time)
```

```
## Iteration: 1
## Iteration: 2
## Iteration: 3
## Iteration: 4
## Iteration: 5
## Iteration: 6
## Iteration: 7
## Iteration: 1
## Iteration: 2
## Iteration: 3
## Iteration: 4
## Iteration: 5
```

```
## Estimated Bayes factor in favor of fit_new over fit_time: 11.90251
```

Which also indicates that our data gives moderate support for the model with a treatment by time interaction over a model with only an effect of time. We are left to conclude that the evidence supports a treatment effect, and that it is highly unlikely to be smaller than 0.3 standardized units on average over the whole observation period. If we were to conduct a replication a more informative prior based on this posterior would be justified.
